# Capacity Building to Improve Hospital Managers’ Performance in West Asia

**DOI:** 10.15171/ijhpm.2019.14

**Published:** 2019-03-06

**Authors:** Leila Doshmangir, Amirhossein Takian

**Affiliations:** ^1^Tabriz Health Services Management Research Center, Iranian Center of Excellence in Health Management, School of Management and Medical Informatics, Tabriz University of Medical Sciences, Tabriz, Iran.; ^2^Social Determinants of Health Research Center, Health Management and Safety Promotion Research Institute, Tabriz University of Medical Sciences, Tabriz, Iran.; ^3^Department of Global Health and Public Policy, School of Public Health, Tehran University of Medical Sciences, Tehran, Iran.; ^4^Health Equity Research Centre (HERC), Tehran University of Medical Sciences, Tehran, Iran.; ^5^Department of Health Economics and Management, School of Public Health, Tehran University of Medical Sciences, Tehran, Iran.

## Dear Editor,

Since 2016, the Ministry of Health and Medical Education (MoHME), in collaboration with the World Health Organization (WHO), has initiated a comprehensive program to empower over 700 public hospital managers in Iran and build their capacity for improving their performance and productivity. This was a response to a national survey of 2015 to assess the competency of public hospital managers in Iran and determine their training needs. The plan aims to meet the requirements of recent Health Transformation Plan to reach Universal Health Coverage by 2025 in Iran.^1^ To start with, seven global scholars along with pioneer national experts, acting as facilitators, developed a core curriculum across seven domains including governance, leadership and strategic management; human resources management; hospital financing; hospital emergency and disaster management; hospital supplies and supportive services; and quality improvement and patient safety; and health information management. The MoHME identified the facilitators and twinned them with international trainers. The implementation began with one week training of 85 Iranian potential trainers in November 2016 in the capital city of Tehran. The trainers were English fluent academics with good practical experience in healthcare management. The MoHME also facilitated a workshop for strengthening trainers’ skills and expertise in various techniques and methods for adult learning and teaching. The next phase was to cascade a tailored training package across ten geographical hubs for all hospital managers nationwide. The hubs were selected from the 67 public medical universities and their affiliated hospitals, with each hub within one of the larger medical universities being responsible for neighboring universities. During the first six months of running the program, until August 2017, 250 hospital managers completed a course of seven modules lasting a total of 28 days (average four full days for each module with one month gap between each module) of rotational training. The quality of training, its challenges and content were assessed by WHO global experts, who recommended more focus on practical parts of the course and use of more national examples for better understanding of the module. The recommendations were applied to improve the training of the second cohort, whose training began in November 2017 and finished in July 2018. Since October 2018, the MoHME has commenced training of national trainers to expand capacity building among hospital leaders, to be delivered during 2019.

Despite fundamental inter-country differences, hospitals in the West Asia region have been suffering from long-standing common challenges, ie, fragmented management, lack of strategic thinking, inefficient leadership, inadequate knowledge and skills on change management, poor supply of medical products especially used to diagnosis and care such as drugs and pharmaceuticals or personal care products, equitable resource allocation, distribution and efficient use of resources, weak health information system, and ineffective monitoring and control strategies.^2-6^

Building upon Iran’s successful experience, the WHO advocated similar initiative to be conducted in other countries in the region. WHO conducted a need assessment survey in collaboration with selected countries. The Iranian MoHME and WHO delivered the first round of international training of trainers for representatives from neighboring counties of Iraq (N = 22) and Afghanistan (N = 25). The course lasted five days and was held in August 2018 at the National Public Health Management Center (NPMC) affiliated to Tabriz University of Medical Sciences, Tabriz, Iran. The Iranian MoHME aims to extend this program to Oman and Jordan in 2019. The international trainers are expected to cascade the modules for hospital managers and other staff in their respective countries.

The ultimate goal of the program is to create a platform to share and learn from each other’s experiences and advance hospital managers’ knowledge and skills to improve hospital performance. Such training courses need to continue and integrated into hospitals’ career development systems, as a necessary means to improve managers’ performance and not an end itself.

Although continuous training is essential to enhance performance, not all components required for organizational capacity building will be achieved through training. Concurrent systematic reforms are needed to improve public hospital performance. These include establishing strategic purchasing by insurance funds, implementation of purchaser-provider split, supply management or developing outsourcing. To avoid a “one size fits all” approach, systematic and evidence-informed approach is essential to assess the capacity gaps and identify local needs of hospital managers and then plan to enhance their competency to improve efficiency, effectiveness and responsiveness of healthcare services.

## Ethical issues

Not applicable.

## Competing interests

Authors declare that they have no competing interests.

## Authors’ contributions

Both authors contributed to the intellectual development of the paper. LD conducted literature review, collected data, and drafted the paper. AT provided written contribution, technical advice on the content and direction of the paper. Both read and approved the final draft.

## Authors’ affiliations

^1^Tabriz Health Services Management Research Center, Iranian Center of Excellence in Health Management, School of Management and Medical Informatics, Tabriz University of Medical Sciences, Tabriz, Iran. ^2^Social Determinants of Health Research Center, Health Management and Safety Promotion Research Institute, Tabriz University of Medical Sciences, Tabriz, Iran. ^3^Department of Global Health and Public Policy, School of Public Health, Tehran University of Medical Sciences, Tehran, Iran. ^4^Health Equity Research Centre (HERC), Tehran University of Medical Sciences, Tehran, Iran. ^5^Department of Health Economics and Management, School of Public Health, Tehran University of Medical Sciences, Tehran, Iran.
